# Radiographic and functional outcomes after displaced intra-articular calcaneal fractures: a comparative cohort study among the traditional open technique (ORIF) and percutaneous surgical procedures (PS)

**DOI:** 10.1186/s13018-016-0426-6

**Published:** 2016-08-22

**Authors:** Carlo Biz, Elia Barison, Pietro Ruggieri, Claudio Iacobellis

**Affiliations:** Orthopaedic Clinic, Department of Surgery, Oncology and Gastroenterology DiSCOG, University of Padua, via Giustiniani 2, 35128 Padova, Italy

**Keywords:** Calcaneal fractures, Percutaneous surgery, ORIF, Böhler’s angle, Sander’s classification

## Abstract

**Background:**

Open reduction with internal fixation (ORIF) and percutaneous surgery (PS) are the most common surgical procedures for the treatment of displaced intra-articular calcaneal fractures. The purpose of this retrospective study was to compare the clinical and radiological results of these techniques and to verify the prognostic value of the radiographic measurement tools proposed in the literature.

**Methods:**

A consecutive series of 104 calcaneal fractures was included in this analysis. Essex-Lopresti and Sanders classifications were used to evaluate the injuries, and their prognostic correlation was tested. Böhler’s angle was measured pre- and postoperatively and evaluated as radiological outcome. Clinical outcomes were evaluated using the American Orthopaedic Foot and Ankle Society hindfoot scale (AOFAS), Maryland Foot Scale (MFS), 17-Foot Function Index (FFI), Short Form-36 (PCS), and a 10-point visual analogue scale (VAS).

**Results:**

A total of 87 fractures (5 bilateral), 54 in males and 28 in females, were evaluated with a mean follow-up of 77.0 months. Overall mean age was 51.6 years old. The most frequent cause of trauma was a fall from a height. According to Essex-Lopresti, there were 58 joint depression fractures, 26 tongue, and 3 comminute. According to Sanders: 37 type II, 31 type III, and 19 type IV. Patients were divided into three groups according to surgical treatment: 19 in the *ORIF group*, 35 in the *PS Screw group*, and 33 in *PS K-wire group*. The ORIF group obtained significantly better results (82 AOFAS, 86 MFS, 19.6 FFI, 46.2 PCS, 8 VAS) with respect to the PS K-wire group (74 AOFAS, 76 MSF, 26.4 FFI, 40.8 PCS, 6 VAS). The PS Screw group obtained intermediate results (79 AOFAS, 82 MFS, 22.4 FFI, 41.6 PCS, 7 VAS). The restoration of the Böhler’s angle was achieved most frequently (*p* = 0.02) in the ORIF group, without better clinical results.

**Conclusion:**

The results were best in the ORIF group, despite its risk of complications, inferior in the PS Screw group, however without statistical significance (*p* > 0.05), and worse in the PS K-wire group. Finally, our data confirmed the prognostic correlation between the two radiographic classifications used and the clinical outcomes.

## Background

Calcaneal fractures are the most frequent injuries of the tarsal bone, with an incidence of 11.5 per 100,000 people per year [1] and account for about 1–2 % of all fractures in the human body [2]. They occur 2.4 times more frequently in males, whose age of incidence has been reported to range between 20 and 29 years [1]. The risk factors have been well described and include osteoporosis, diabetes, autoimmune disorders, and increased age [3]. More than 70 % of calcaneal fractures are intra-articular, involving the subtalar joint, mostly caused by a fall from a height with the heel directly hitting the ground [4].

Their management remains controversial [5], mainly due to a low level of evidence. Nowadays, a moderate consensus tends toward Sanders computerized tomography (CT) classification [6] and the Böhler angle measurement [7] as useful diagnostic and management tools. Further, some authors [8, 9] have suggested conservative treatment, even for displaced intra-articular calcaneal fractures, which has continued to have poor results [10–12]. Currently, open anatomic reduction and internal fixation through the extended lateral approach is considered the most widely accepted surgical technique for displaced intra-articular calcaneal fractures [13]. This approach permits wide visualization of the subtalar joint and anatomical reduction and provides a sufficient lateral area for rigid fixation. However, the use of plate fixation has been correlated to deep infection in reported rates of 8 to 25 %, with superficial infection occurring in less than 40 % and wound necrosis in approximately 14 %, mainly due to the thinness of superficial soft tissue [10, 14–18]. In these cases, additional open surgery for removal of the metal work, often associated with surgical debridement and free flap coverage, is required [19, 20]. These complications delay rehabilitation, and hospitalization, absence from work and recreation can be very long when a second surgery is necessary [21].

Hence, the significant risk of open surgery has driven surgeons to look for less traumatic techniques, including closed reduction, external fixation, and percutaneous surgery (PS), in order to reduce complication rate and hospitalization time [22–24]. Several authors have reported that a minimally invasive approach can improve radiographic parameters and can be used to achieve satisfactory results with less early postoperative pain, better range of motion, higher functional scores of the injured ankle, and fewer complications compared with open surgery [25, 26]. Moreover, these procedures, using singular or combined percutaneous screws and Kirschner wire (K-wires), can be performed sooner, as swelling is not a contraindication, to minimize blood loss and soft tissue trauma. For these reasons, percutaneous surgery is indicated also on elderly patients and those with systemic comorbidities [27].

The primary aim of the present retrospective study was to investigate, evaluate, and compare the clinical and radiographic outcomes among open reduction and internal fixation (ORIF) and PS approaches for the treatment of displaced intra-articular calcaneal fractures Sanders II-IV. We performed a retrospective analysis to compare patients who underwent the ORIF technique with plate fixation with those who underwent PS with screws or K-wire fixation. Secondly, during the investigation, we intended to verify the real prognostic value of the most known classification tools proposed for these fractures and the most used radiological outcome measurements described in the literature to assess the subtalar articular surface.

## Methods

### Patients

In this retrospective cohort study, we examined clinical and radiographic data from a consecutive series of patients with diagnosis of displaced intra-articular calcaneal fractures. At our level I healthcare trauma center, from January 2006 to December 2013, these patients underwent one of the following surgical procedures: (a) open reduction and internal fixation (ORIF) or (b) PS by stabilization with cannulated screws or Kirschner wires. All subjects participating in this study received a thorough explanation of the risks and benefits of inclusion and gave their oral and written informed consent to publish the data. The study was performed in accordance with the ethical standards of the 1964 Declaration of Helsinki as revised in 2000.

### Inclusion and exclusion criteria

The inclusion criteria were the diagnosis of a closed or open displaced intra-articular calcaneal fracture with two or more millimeters displacement (Sanders type II–IV), treated either with ORIF or PS by stabilization with cannulated screws or Kirschner wires. All patients considered in this study had to be between 18 and 85 years of age and give their informed consent to participate. Specific patient exclusion criteria included a history of severe neurological deficits, previous foot surgery or trauma, primary arthrodesis or amputation, Gustilo grade III open fractures, diagnosis of rheumatological diseases or psoriatic arthritis, foot neuropathy, severe vascular insufficiency, and alcohol or drug abuse. Patients having secondary arthrodesis were included, as this is considered as a complication of first treatment.

### Surgical techniques

All operative procedures were performed by one of the two surgeons, the senior authors (C.I. and C.B.). The treatment choice among the two surgical techniques was based on the preferences and experience of the surgeons involved in the operations and on the basis of the soft tissue condition of the single cases. Prophylactic cefazolin (2 g) was administered and continued 24 hours after surgery. Postoperative antithrombotic therapy (Natrium Enoxaparin) was given until weight bearing. In all procedures, a plexus anesthesia was performed consisting in a regional block, which involved both sciatic and femoral nerves (bi-block). Sedation was used when necessary. A thigh tourniquet was always applied only when the ORIF technique was performed.

### ORIF technique

Open surgery was performed only in patients without soft tissue damage after the swelling had subsided and skin wrinkles were present. The patient was placed in the lateral decubitus position on a radiolucent operating table, with the foot elevated on an appropriate support. A full-thickness “L”-shaped lateral incision was used with a gentle curve between the two segments. The fracture was reduced and temporarily fixed with K-wires under radiographic guidance. When the reduction was satisfactory, as seen with the radiographic intensifier, final stabilization was obtained with a low-profile plate (Synthes calcaneal locking plate) and titanium angle-stable screws. In some cases, injectable graft substitute (Norian SRS skeletal repair system) was used to fill the bony defect beneath the articular surface. A compression dressing was applied on the operated side for 48 h after surgery. Patients were kept non-weight-bearing with two crutches for 8 weeks, while passive and active ankle range of motion (ROM) exercises were allowed 15 days after plating fixation.

### Percutaneous surgery with screw or K-wire fixation

During PS, the patient was placed prone on a radiolucent operating table, with the foot protruding. In joint depression fractures, a small lateral incision permitted the fragments to be reduced using a periosteotome under fluoroscopic guidance. To achieve reduction, under image intensifier control, one or two 2-mm K-wires were inserted from the calcaneal tuberosity toward the subtalar joint. Then, during closed reduction, using the K-wires like a joystick by external maneuvres and a leverage technique with axis stress onto the pins down the distal side, restoration of Böhler’s angle was attempted. Fluoroscopic images in lateral and axial radiographic views allowed the evaluation of the anatomical reduction. Final stabilization was obtained with other 2-mm K-wires or with titanium cannulated screws (Synthes, 6.5 mm diam.), inserted in the same posterior-anterior direction. In some cases, for better support of the thalamic region, a latero-medial screw was introduced through the incision used for the talar joint reduction. Screw fixation was performed being careful to avoid the protrusion of the screw head. A compression dressing was applied on the operated side for 48 h after surgery. Patients were kept non-weight-bearing for 4 weeks during which passive and active ankle ROM exercises were allowed 15 days after screw fixation or 30 days when K-wires were used.

### Patient assessment

Data collection, as well as radiological and clinical evaluation, was performed at our institution during a period of 11 months, from March 2015 to Jenuary 2016, by an external and independent investigator, the junior author (E.B.), not involved in the patients’ treatment.

Patients’ characteristics (gender, age at trauma, body mass index (BMI), comorbidities, American Society of Anesthesiologists (ASA) class to globally estimate surgical risk [28], smoking habits), trauma characteristics (affected side, mechanism, open fractures, concomitant injuries), and treatment characteristics (open or closed approach, implant type, duration of surgery, hospitalization, complications) were collected from the electronic database of the hospital. Finally, the patients included in this investigation were divided into three groups according to the fixation method used:

1. *ORIF group*
2. *PS Screw group*
3. *PS K-wire group*

### Radiographic outcome measures

Radiographic data were obtained by reading the radiographic computerized images, available in the computer system of our institute. The radiographic evaluation comprised the analysis of conventional radiographs, including lateral, axial, and internal oblique views in the preoperative, postoperative, and follow-up periods and preoperative CT scans. The fractures were classified according to Essex-Lopresti [8] and Sanders et al. [6]. Böhler’s angle was measured from the trauma, postoperative, and follow-up radiographs. The restoration after surgery of a physiological range of Böhler’s angle (20°–40°) [29] was considered a good radiological outcome. All radiological evaluations were performed with the Med Station program (the X-ray data base of our hospital). This software allows the retrieval of electronically computer-assisted measurements from radiographs. In particular, we used a diagnostic liquid crystal display (LCD) CORONIS 5MP display (produced by Barco, Rome, Italy) as viewing monitor to analyze the fractures and their outcomes. Finally, examination of lateral, axial, and internal oblique view radiographs at different follow-ups during the evaluation of patient outcome, showing complete bridging bone/callus formation and the absence of radiolucent lines, was used to define bone healing.

### Functional outcome measures

At the time of this study, a phone contact was attempted for all patients who met inclusion criteria, and a follow-up appointment was fixed. Patients who returned were examined, and clinical results were measured with validated questionnaires. To quantify pain and functional disability, the American Orthopaedic Foot and Ankle Society (AOFAS) hindfoot scale [30], the Maryland Foot Score (MFS) [6], and the 17-Foot Functional Index (FFI) [31] were used. The first includes 9 questions related to pain (1 question; 40 points), function (7 questions; 50 points), and alignment (10 questions; 10 points). A healed patient without any problems could reach 100 points. The second is a scoring system conceptually analogous to AOFAS, but points are differently distributed: 45 for pain and 55 for functional limitation. The third measures the persistence of pain, disability, and restriction of activity, with 17 number rating scales from 0 to 10. The maximum score is 100, which indicates complete disability. All patients were also asked to complete the Short Form 36 (SF-36) [32], which is a validated questionnaire widely used for different pathologies to measure the patient-reported quality of life. It consists of 36 questions, representing 8 health domains that are combined into physical (PCS) and mental component summaries (MCS), using the US population as reference. For this analysis, both summary scores were used. Further, a 0–10 visual analogue scale (VAS) was used to quantify patient satisfaction of the results, where 0 means maximum dissatisfaction and 10 full satisfaction. The patients were also queried regarding shoe-related problems, work and sports activities at the age of the trauma, and their resumption. In particular, hindfoot inversion and eversion mobility was evaluated by dividing patients into four categories of stiffness: absent, mild, moderate, and severe. Finally, during the analysis, any complications were recorded and also their distribution per surgeon.

### Statistical analysis

Statistical analyses were performed by an independent statistician from the Department of Statistics at the University of Padua, using SAS 9.2 (SAS Institute Inc., Cary, NC, USA) for Windows. Continuous data were checked for a normal distribution with the Shapiro-Wilk test and expressed with average and standard deviation or median and minimal-maximal value. Outcome results were compared among groups with different surgical fixation, Sanders classifications, Essex-Lopresti classification, and restoration of Böhler’s angle. The chi-square or Fisher’s exact tests for categorical variables and analysis of variance or the Kruskal-Wallis test for continuous variables was used. In the case of more than two groups, continuous variables were compared in pairs with Dunn’s test. The correlation between trauma and patient characteristics and the onset of complications was tested with univariate logistic regression; the odds ratio and its 95 % confidence interval was calculated. A *p* value of <0.05 was taken as the threshold of statistical significance.

## Results

### Patient data

During a 7-year period, 98 patients with 104 fractures (6 were bilateral fractures) were treated at our institution. We could not evaluate 16 patients (17 fractures) as 6 refused to participate, 3 were dead at the time of evaluation, and for 7 subjects, a follow-up address could not be retrieved. Hence, 82 patients were retrospectively enrolled in the present case series study, 5 with bilateral fractures, for a total of 87 fractures operated (83.6 % of the total), and all of these patients underwent clinical and radiographic assessment at the final follow-up. The patients’ details are summarized in Table 1. There were 54 men (2 bilateral cases for a total of 56 fractures, 64.4 %) and 28 women (3 operated bilaterally, 31 fractures, 35.6 %). Overall mean age at the time of injury was 51.5 years old (±15.7 years). The average follow-up period was 77.0 (±30.0) months, i.e., more than 6 years.

**Table 1:** Characteristics of patients and fractures

	Overall (*n* = 87)	ORIF (*n* = 19)	PS Screws (*n* = 35)	PS K-wires (*n* = 33)
Male gender^a^	54 (62.0 %)	11 (57.9 %)	24 (68.6 %)	19 (57.6 %)
Age	51.5 (±15.7)	45.8 (±12.5)	51.4 (±15.9)	54.8 (±16.7)
Left side	46 (52.9 %)	10 (52.6 %)	15 (42.9 %)	21 (63.6 %)
BMI	25.2 (±4.0)	24.1 (±4.1)	25.8 (±3.9)	25.1 (±4.1)
Comorbidity				
Cardiovascular	8 (9.2 %)	1 (5.3 %)	2 (5.7 %)	5 (15.1 %)
Diabetes	8 (9.2 %)	0	4 (11.4 %)	4 (12.1 %)
Hypertension	15 (17.2 %)	3 (15.8 %)	5 (14.3 %)	7 (21.2 %)
Smoker^a^	24 (29.3 %)	7 (38.9 %)	12 (36.4 %)	5 (16.1 %)
ASA^a^				
1	47 (57.3 %)	11 (61.1 %)	21 (63.7 %)	15 (48.4 %)
2	32 (39.0 %)	7 (38.9 %)	11 (33.3 %)	14 (45.1 %)
3	3 (3.7 %)	0	1 (3.0 %)	2 (6.4 %)
Mechanism^a^				
Fall from height	61 (74.4 %)	17 (94.4 %)	23 (69.7 %)	21 (67.7 %)
Low energy trauma	15 (18.3 %)	0	7 (21.3 %)	8 (25.9 %)
Road accident	6 (7.3 %)	1 (5.6 %)	3 (9.0 %)	2 (6.4 %)
Concomitant injuries				
Lower limb	21 (24.1 %)	4 (21.0 %)	10 (28.5 %)	7 (21.2 %)
Spinal cord	15 (17.2 %)	4 (21.0 %)	5 (14.2 %)	6 (18.1 %)
Upper limb	2 (2.3 %)	0	1 (2.8 %)	1 (3.0 %)
Exposition	5 (5.7 %)	0	2 (5.7 %)	3 (9.0 %)
Days before surgery	6 (0 to 25)	7 (1 to 20)	3 (0 to 18)	8 (0 to 25)
Surgical duration	58 (15 to 240)	105 (70 to 190)	60 (25 to 240)	35 (15 to 180)
Hospitalization	9 (2 to 123)	15 (7 to 37)	6 (2 to 27)	13 (4 to 123)
Complication or second surgery	23 (26.4 %)	9 (47.4 %)	8 (22.9 %)	6 (18.2 %)
Sanders				
2	37 (42.5 %)	8 (42.1 %)	14 (40.1 %)	15 (45.4 %)
3	31 (35.7 %)	10 (52.7 %)	12 (34.2 %)	9 (27.3 %)
4	19 (21.8 %)	1 (5.2 %)	9 (25.7 %)	9 (27.3 %)
Lopresti				
Joint	58 (66.7 %)	13 (68.4 %)	22 (62.8 %)	23 (69.8 %)
Tongue	26 (29.9 %)	6 (31.6 %)	12 (34.3 %)	8 (24.2 %)
Comminuted	3 (3.4 %)	0	1 (2.9 %)	2 (6.0 %)
Böhler pre	17.5° (−34.6 to 49.5)	20.8° (−7.2 to 49.5)	16.7° (−16.8 to 37.4)	17.2° (−34.6 to 37.3)

^a^ Bilateral cases were considered one time

The most frequent trauma mechanism was a fall from a height in 61 cases (74.4 %). Other causes were low-energy trauma (LET) in 15 cases (18.3 %), such as falling from less than 1 m, falling from standing or direct foot trauma, and road accidents in 6 cases (7.3 %). Concomitant fractures interested lower limbs in 21 cases (24.1 %), the spinal column in 15 (17.2 %), and upper limbs in 2 (2.3 %). Open fractures were 5 (5.7 %): 3 Gustilo grade I and 2 grade II. Patient comorbidities and risk factors were recorded as well: mean BMI was 25.2 kg/m^2^ (±4.0) and 9 subjects were obese (BMI >30); active smokers were 24 (29.3 %); 15 patients (17.2 %) reported hypertension, 8 (9.2 %) diabetes, and 8 (9.2 %) heart disease (previous myocardial infarction, arrhythmias, valvular disease) or vascular disease. According to ASA classification to globally estimate surgical risk, there were 47 patients ASA 1 (57.3 %), 32 ASA 2 (39.0 %), and 3 ASA 3 (3.7 %).

On average, 10.8 operations were performed every year for calcaneus osteosynthesis: 41 operations on the right foot and 46 on the left. Of the 87 surgical procedures, 55 (63.3 %) were performed by C.I. and 32 (36.7 %) by C.B.; 19 (21.8 %) were performed using the ORIF technique (the ORIF group) and 68 (78.2 %) by PS, using two or three screws in 35 (40.2 %) percutaneous approaches (the PS Screw group) and K-wires in 33 (37.9 %) cases (the PS K-wire group). Time between trauma and surgery ranged from 1 to 25 days with a median time of 6 days. The mean duration of surgery was 105 min for the ORIF group, 60 min for the PS Screw group, and 35 min for the PS K-wire group.

### Radiographic outcomes

**Fig. 1:**
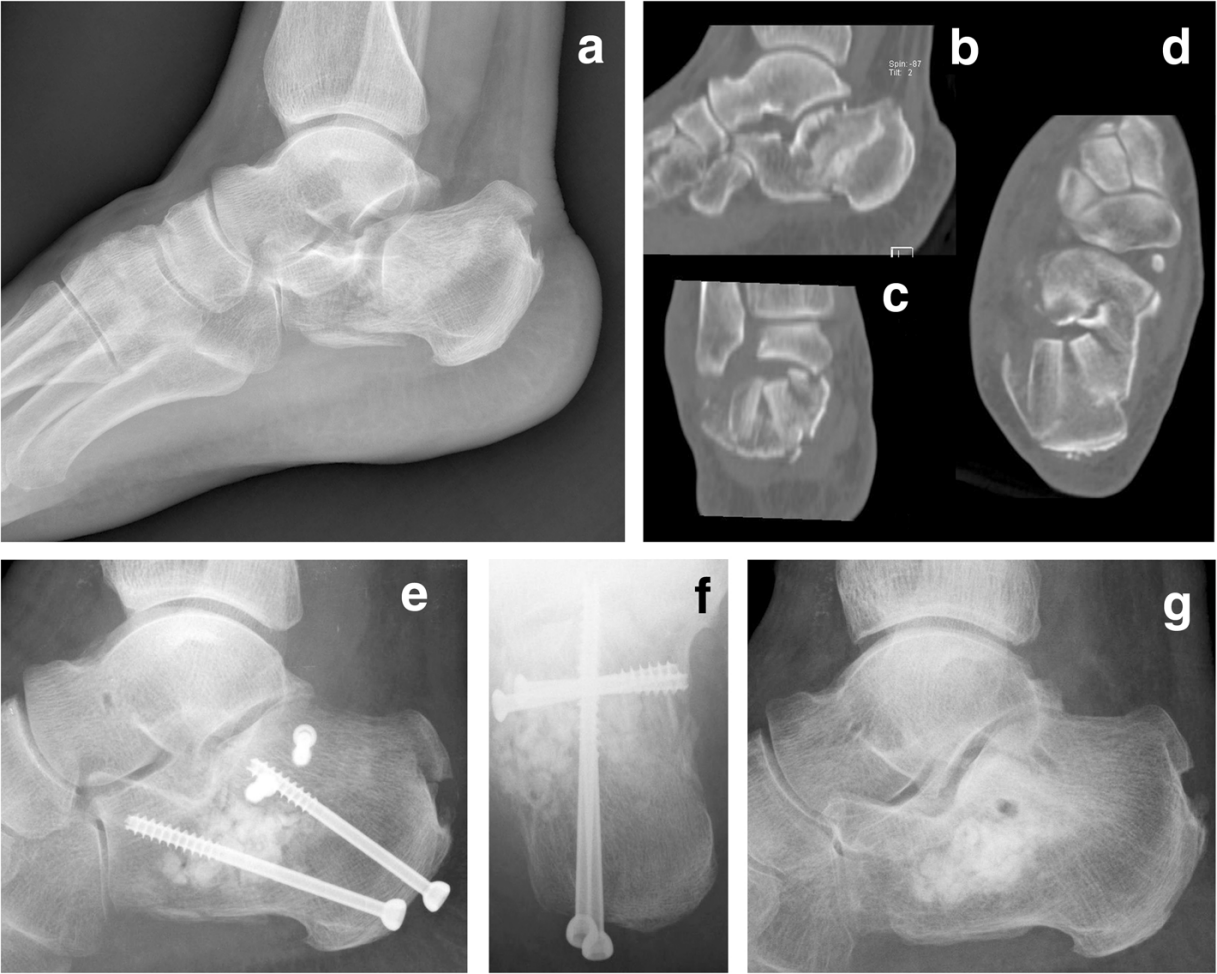
Male 62 years old: (**a–d**) preoperative X-ray and CT; (**e–f**) 30 days post op.; (**g**) follow-up 1 year later

**Fig. 2:**
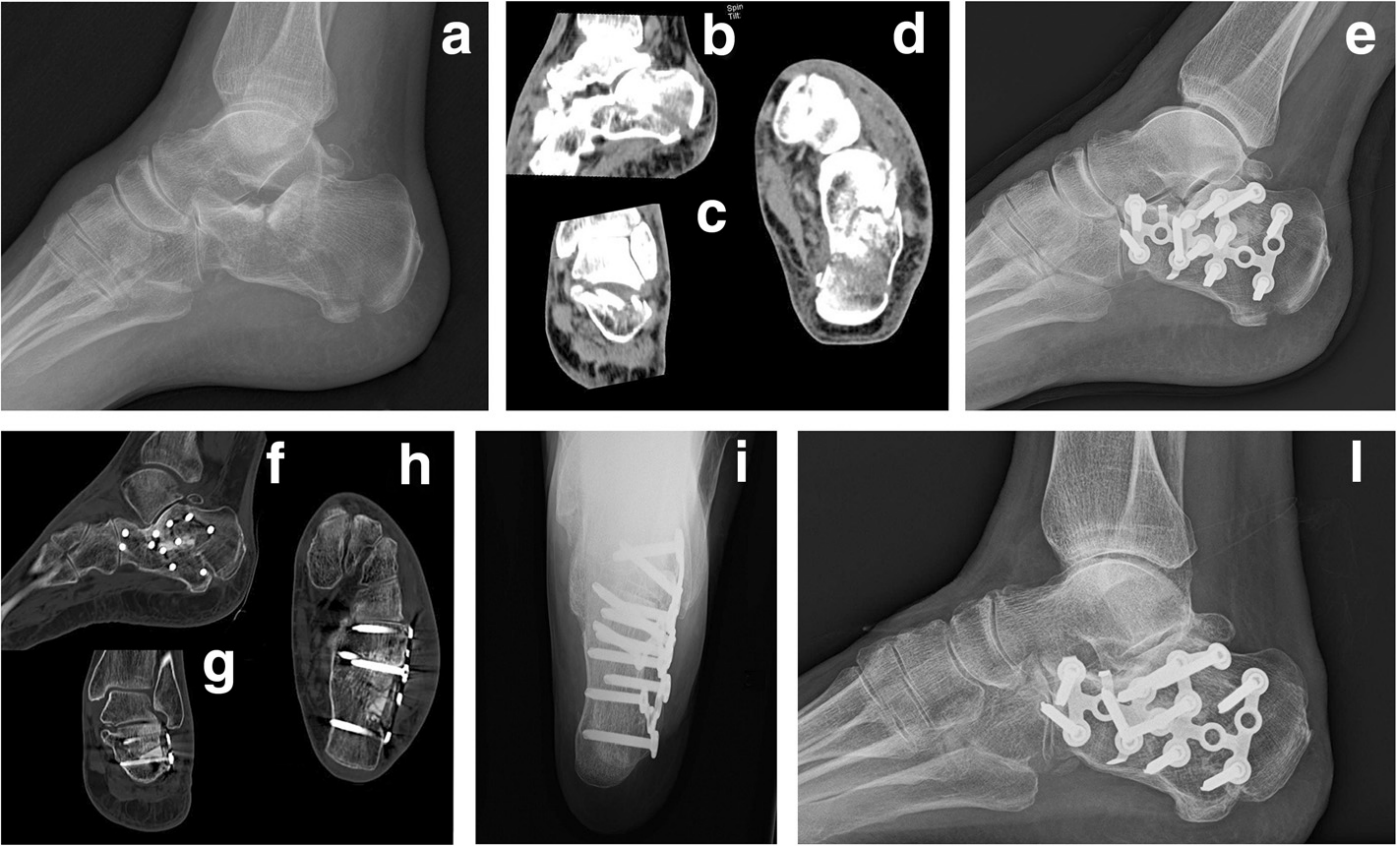
Female 56 years old: (**a–d**) preoperative X-ray and CT; (**e**) postoperative check up; (**f–h**) follow-up 10 months later; (**i–l**) follow-up 2.5 years later

Preoperative radiological images were analyzed, and the fractures were classified using the two classification systems (Figs. 1 and 2). According to Essex-Lopresti, 58 (66.7 %) were joint depression fractures and 26 (29.9 %) were tongue. Comminuted fractures, not classifiable with Essex-Lopresti, were found in three patients (3.4 %). According to Sanders et al., there were 37 (42.5 %) type II fractures, 31 (35.7 %) type III, and 19 (21.8 %) type IV. On the lateral preoperative radiographs, the mean Böhler’s angle was 17.9°. However, in 40 cases (45.9 %), a normal range (20°–40°) was found. This angle was measured also after surgery and was restored within normal range in 15 ORIF procedures (78.9 %), 12 PS screw fixations (34.3 %), and 16 PS K-wire fixations (48.5 %), with mean improving values of 29° (range 8.2°–37.6°), 17° (range −12.8°–39.1°) and 21.5° (range 18.4°–48.6°), respectively. ORIF permitted significantly more frequent (*p* = 0.02) restoration to a normal angle. The radiological consolidation of the fracture occurred after a mean period of 3.4 months (range 1.7–7). The radiographic outcomes of our cohort are summarized in Table 2.

**Table 2:** Clinical and radiological outcomes

	Overall (*n* = 87)	ORIF (*n* = 19)	PS Screws (*n* = 35)	PS K-wires (*n* = 33)	*p* value
Follow-up months	77.0 (±30.0)	78.7 (±21.7)	71.1 (±32.8)	82.2 (±28.1)	
Böhler post	20.9° (−18.4 to 48.6)	29.0° (8.2 to 37.6)	17.0° (12.8 to 39.1)	21.5° (−18.4 to 48.6)	
Normal Böhler (20°–40°)	43 (49.4 %)	15 (78.9 %)	12 (34.3 %)	16 (48.5 %)	**0.029**
VAS	7 (3 to 10)	8 (6 to 10)	7.5 (5 to 10)	6.5 (3 to 10)	**0.004** ^*^
AOFAS	80 (25 to 100)	82 (66 to 97)	79.5 (57 to 100)	76 (25 to 93)	**0.035** ^*^
MSF	82 (29 to 99)	87 (76 to 99)	82.5 (57 to 99)	77.5 (29 to 94)	**0.007** ^*^
FFI	22.4 (1.7 to 58.2)	20.5 (2.9 to 30)	21.7 (2.3 to 55.8)	24.6 (1.7 to 58.2)	0.420
SF-36 PCS	42.3 (24 to 56.8)	46.4 (28.9 to 56.8)	41.6 (24 to 54.2)	41.8 (25.3 to 51.9)	**0.019** ^*^
SF-36 MCS	50.5 (34.2 to 62.2)	50.4 (37.5 to 6.5)	51.4 (38.9 to 62.3)	50.6 (34.2 to 58.8)	0.374
Work resumption^a^	41 (65.0 %)	14 (82.3 %)	15 (65.2 %)	12 (52.2 %)	0.185
Sport resumption^b^	11 (57.9 %)	3 (27.3 %)	6 (54.5 %)	2 (18.2 %)	0.494
Walking barefoot	69 (79.3 %)	19 (100 %)	26 (74.3 %)	24 (72.7 %)	0.520
Running	22 (25.2 %)	5 (26.3 %)	9 (25.7 %)	8 (24.2 %)	0.866
Rigidity					0.340
Absent/mild	46 (52.8 %)	8 (42.1 %)	22 (62.9 %)	16 (48.5 %)	
Moderate/severe	41 (47.2 %)	11 (57.9 %)	13 (37.1 %)	17 (51.5 %)	
Shoes change	43 (49.4 %)	9 (47.3 %)	16 (45.7 %)	18 (54.5 %)	0.577

^*^ Pair comparison confirmed statistical significance differences between ORIF and PS K-wires groups

Bold character indicate significant values (*p* < 0.05)

^a^ Workers at the time of fracture were 63

^b^ Sportive at the time of fracture were 19

### Clinical outcomes

The clinical outcomes, subdivided between surgical groups, are summarized in Table 2. Statistical analysis with pair comparison showed significant differences between the results of the ORIF and PS K-wire groups measured with the scales AOFAS, Maryland Foot Score (MSF), VAS and SF-36 PCS. In the entire population, the AOFAS scale measured excellent results (90–100 points) in 11 patients (12.6 %), good results (75–89 points) in 46 patients (52.9 %), fair results (50–74 points) in 26 patients (29.9 %), while 4 (4.6 %) fractures were graded as failures (<50 points). Similarly, the MFS scale showed excellent results (90–100 points) in 13 patients (14.9 %), good results (75–89 points) in 50 (57.5 %), fair results (50–74 points) in 15 (21.1 %), and there were 4 (4.6 %) failures (<50 points). With the FFI questionnaire, 13 patients (14.9 %) obtained optimal scores lower than 10; in 49 cases (56.3 %), the scores were between 10 and 30; in 21 cases (24.1 %), between 30 and 50; and in 4 (4.6 %), scores were higher than 50. Only 18 patients (20.7 %) were completely satisfied with a VAS score of 9–10: 8 (42.1 %) in the ORIF group, 6 (17.1 %) in the PS Screw group, and 4 (12.1 %) PS K-wire group.

In our sample, only 19 patients (22.9 %) had practiced sports regularly before injury. At the last follow-up, 11 (57.9 %) had returned to their sports activities. Among them, 3 (27.3 %) were of the ORIF group, 6 (54.5 %) of the PS Screw group, and 2 (18.2 %) of the PS K-wire group. Irrespective of the surgical procedure, less than 30 % of the subjects were able to run, with no differences among the 3 groups. All of the ORIF group patients were able to walk barefoot, as well as 26 (74.3 %) of the PS Screw group and 24 (72.7 %) of the PS K-wire group. With regard to hindfoot inversion and eversion mobility, we found absent to mild stiffness in 8 subjects of the ORIF group (42.1 %), 22 of the PS Screw group (62.9 %), and 16 of the PS K-wire group (48.5 %). Moderate to severe stiffness was present in 11 patients of the ORIF group (57.9 %), 13 of the PS Screw group (37.1 %), and 13 of the PS K-wire group (51.5 %). No significant differences were found upon statistical analysis (*p* = 0.34). A total of 43 patients (49.4 %), without substantial differences between treatment groups, reported that it was impossible to wear the same shoes used before the trauma, or there were some restrictions on usable shoe shape. This condition was significantly higher (*p* = 0.001) in females, with 21 of 31 cases (67.7 %), than in males, with 18 of 56 cases (32.1 %).

### Complications

Among 87 fractures treated, 23 (26.4 %) calcanei experienced an adverse event or needed a second surgery. This happened more frequently in the ORIF group (*n* = 9; 47.4 %) than in the PS Screw (*n* = 8; 22.9 %) and K-wire groups (*n* = 6; 18.2 %). This difference was at the limit of significance (*p* = 0.058). The complication rate appears equally distributed between the two surgeons involved in the series: C.I. reported 14 cases (25.4 %) and C.B. 9 (28.1 %). The most frequent complications involved soft tissues. There were 10 cases (11.5 %) of wound dehiscence or necrosis: 5 (26.3 %) in the ORIF group, 2 (5.7 %) in the PS Screw group, and 3 (9.1 %) in the PS K-wire group. Daily medications were sufficient to resolve 5 cases (5.7 %), while 2 (2.3 %) surgical debridements and 3 (3.4 %) free flap transplantations were necessary in the other cases. In 4 cases (4.6 %), there was deep infection, 3 (15.8 %) in the ORIF group, and 1 (2.8 %) in the PS Screw group. To resolve these complications, metal work removal was performed. Secondary arthrodesis was performed in 4 cases (4.6 %) because of the persistence of constant pain, 3 (8.6 %) in the PS Screw group, and 1 (3.0 %) in the PS K-wire group. Furthermore, 5 patients (5.7 %), which complained of persistent pain and signs of osteoporosis, developed Sudeck syndrome. Other 3 cases (3.4 %) developed nerve complications with persistent dysesthesia or hypoesthesia. The correlation between complications and patient or trauma characteristics was analyzed with logistic regression. Only Sanders III fractures had an increased risk of complication, with an odds ratio of 4.5 (1.4–14.9).

## Discussion

Calcaneal fractures in males and females are different for the age at trauma and its mechanism. Mitchell et al. [1], in their epidemiological study, detected two frequency peaks at 20 and 50 years of age. The majority of patients were males who had fallen from a height. Female fractures become predominant over 70 years old, typically due to LET. In our cohort, the higher incidence was reached between 50 and 59 years; at older ages, male fractures decrease, while female fractures rise. In our study, LET as mechanism of injury is significantly higher among females (*p* = 0.012), particularly above 60 years old. The observation of Mitchell et al. [1], who identified high-risk jobs and alcohol abuse in males and postmenopausal osteoporosis in females as major causes of calcaneal fractures, appears justified. In our series, there were several complex cases: 25 % of patients presented other lower limb fractures, 18 % of cases presented vertebral fractures and in 5 % cases, the calcaneal fractures were open. The traumas caused by attempted suicide (6.1 %) and outcomes in this cases may be influenced by depression and poor compliance of patients [33, 34]. A large number of fractures, 25.6 %, occurred during work activities. In such cases, some authors reported lower satisfaction and worse results in patients who looked for worker’s compensation [10, 35].

With regard to functional outcome measures, the SF-36 questionnaire obtained a mean physical component score of quality of life (PCS) of 42.3, lower than the reference value of the healthy population (50), but in agreement with the results of several studies [8, 9, 36]. In the literature, similar PCSs were measured in patients with major chronic diseases such as myocardial infarction (42.6), rheumatoid arthritis (43.1), and chronic lung diseases (42.3) [37]. This result means that calcaneal fractures seriously affect health for many years after the trauma. This observation is confirmed by the three foot-specific questionnaires used (AOFAS, MFS, and FFI). The majority of patients, about 80 %, obtained good or fair results, and only 12.6 % (AOFAS) or 14.9 % (MFS and FFI) of patients obtained an optimal score and reported a normal life without any disability. Failure results with persistent severe disability were few, only 4.6 % (AOFAS, MFS, and FFI). Hence, our analysis confirms that the most frequent long-term result of a calcaneal fracture is the persistence of disability and pain for many years after the trauma, but not enough to significantly affect the activities of daily life. Our conclusions are confirmed by those reported in other studies [38].

We observed that in some cases, also non-optimal results were judged satisfactory with the VAS scale if the patient was conscious of the seriousness of his trauma and of the impossibility to eliminate the consequences of it. Instead, only 20.7 % of subjects enrolled were completely satisfied.

Concerning the surgical treatments used in our case series, percutaneous surgery (PS 78 %) was the most frequent. No significant differences were found between populations treated with ORIF, screws, and K-wires, but it is interesting that among the ORIF patients, there were no diabetics, neither were there open fractures, and there was only one Sanders IV classified case. This observation reveals that open surgery was considered only in cases with low preoperative risk, as other authors [6, 21, 27] have suggested. The PS K-wire group obtained significantly lower values (*p* < 0.05) compared with the ORIF group in terms of satisfaction (VAS), quality of life (SF-36 PCS), and functionality (AOFAS, MFS). The comparison between the ORIF group and the PS Screw group was not significant (*p* > 0.05). The FFI questionnaire agreed with other scales but did not show significant differences among the three groups.

We observed that the best functional and radiographic outcomes were obtained in the ORIF group, in accordance with the authors who consider this procedure the gold standard for calcaneal fractures [13, 38, 39]. However, this technique is frequently criticized for its high frequency of complications [35, 38]. Our patients had an overall complication rate of 26.4 %, which is a lower percentage than other studies reported [20, 40]. These results are probably due to the prevalent use of PS in our cohort, which obtained a lower rate of complications: 22.8 % in the PS Screw group and 18.1 % in the PS K-wire group. In the ORIF group, there were complications in 47.3 % of cases; this difference has a borderline statistical significance (*p* = 0.0587). The most frequent adverse events affecting soft tissue (26.3 %) was wound dehiscence in the ORIF group. This percentage is similar to that reported by different authors: 24 % De Groot et al. [38], 24 % Koski et al. [20], and 16 % De Boer et al. [41]. Although some studies have shown a correlation between complication and obesity [35, 42, 43], diabetes, absence of drainage, and complicated fractures [21], in our series, only Sanders III fractures showed a higher risk (odds ratio (OR) = 4.51).

With regard to complications, implants were removed in the ORIF group in 5 cases (26.3 %), 3 (15.8 %) of them for deep infections. This percentage is lower than that reported by other authors, varying from 39 to 49 % [41, 44, 45], while DeWall et al. [46] reported implant removal in 12 % of cases. Screw removal was performed in 18 cases (51.4 %) due to the protrusion of their heads from the bone profile and the resulting discomfort when wearing shoes. Secondary arthrodesis was performed in 4 cases (4.6 %), a percentage lower than those reported in other series (Makki et al., 10.6 % [47]; De Boer et al., 20 % after PS and 33 % in ORIF [41]; Sanders et al., 28.7 % [48]). The low percentage is due to patients’ reluctance to undergo joint fusion.

Patients who had worked before their trauma resumed work in 82.3 % of cases in the ORIF group, 65.2 % in the PS Screw group, and 52.2 % in the PS K-wire group. This difference was not statistically significant (*p* = 0.1856), as the number of patients who had worked before their trauma was low (*n* = 63). De Groot et al. [38] also reported 80 % of ORIF patients resuming their work.

About the reliability of the radiographic tools used to analyze outcomes, Isaac et al. [29] reported a strong correlation between the measurement of a Böhler’s angle less than the normal range and the diagnosis of calcaneal fracture. This observation is very different from the results of our study in which 45.9 % of fractures had an angle greater than 20°. In our series, complete fracture reduction was significantly more frequent in the ORIF group (*p* = 0.02); consequently, only the ORIF technique allowed us to obtain a normal range of Böhler’s angle after surgery in most of the patients (78.9 %). On the other hand, PS rarely restored normal geometry (34.3 % in the PS Screw group; 48.5 % in the PS K-wire group). None of the considered clinical outcome scales (AOFAS, MFS, FFI, PCS, and VAS) showed statistically significant differences (*p* > 0.05) among patients with or without the restoration of normal Böhler’s angle. For this reason, this measurement appears to be an inappropriate radiological outcome. Indeed, some authors have recently proposed using a CT examination as radiological outcome, considering good results only cases without irregularities higher than 3 mm on the articular surface [24, 48]. On the contrary, our data confirmed the prognostic value of the Essex-Lopresti classification: better clinical results (*p* < 0.05) were obtained in tongue type fractures than in joint depression type (median AOFAS 82.5 and 78; MFS 86 and 81; VAS 8 and 7, respectively). This result agrees with the study published by Tornetta [49] that reported good clinical outcomes with PS only in patients with tongue fractures. The prognostic value of today’s most frequently used Sanders classification [50] has been recently validated by the same author comparing long-term clinical results [48]. That study was confirmed by ours; each considered variable obtained better values in the Sanders II group, and statistical analysis showed significantly lower AOFAS, MFS, PCS, and VAS scores comparing Sanders IV and both of the other two classes (*p* < 0.05).

Several potential limitations may influence the results of our study: first its retrospective nature and the different sizes among ORIF and PS groups. We are also aware that only one Sanders IV fracture is present in the ORIF Group, a type of fracture for which it is difficult to achieve good results. Although some authors [6, 51] suggest a primary arthrodesis for the first and definitive surgical approach to these complicated and often hopeless, comminute fractures, we think that they should be treated primarily by percutaneous surgical procedures, exploiting the good vascularization and excellent bone regeneration of the calcaneus [52]. Only secondarily, in case of failure, is it necessary to proceed to a subtalar fusion to treat sequelae. This is the main reason that most of these challenging fractures included were treated percutaneously at our institution. However, in our series, the results were “slightly better” in the ORIF group than the PS Screw group but without statistical significance.

Further, it would have been useful to compare the functional outcomes also with a non-operatively treated group of patients during the same period of recruitment. This would have allowed us to better quantify surgical advantages and disadvantages compared to a conservative treatment option, which is still a controversial subject in the literature [8, 9, 11, 53, 54]. Finally, radiographs analyzed in our study were taken for clinical follow-up and not for research purposes in a strict standardized manner. Hence, this aspect may affect the different projections and altered the radiographic measures.

## Conclusions

In summary, this retrospective and comparative study has shown that the majority of patients who had suffered a displaced intra-articular calcaneal fracture manifested pain and disability several years after trauma, but not enough to significantly affect activities of daily life.

In particular, our data show the ORIF group patients presenting overall superior radiographic and functional outcomes compared to the two other groups. However, as reported in the literature, the results confirm that this treatment strategy is characterized by a high rate of complications, mainly related to surgical wound dehiscence and infections. Hence, we strongly believe that ORIF has to be planned only when swelling and fracture blisters are completely resolved. The patients’ outcome measures of the PS Screw group were inferior to those obtained by the ORIF group, however without any statistical significance (*p* > 0.05). Further, this percutaneous approach minimized wound-related complications and allowed a shorter operative time and hospitalization period. On the contrary, the PS K-wire group patients obtained significantly the worst results of the three groups (*p* < 0.05) in most of the analyzed variables. Hence, we think that this procedure should be avoided except in the case of severe soft tissue damage or open fractures.

Finally, the comparison between radiological measurements and clinical outcomes showed that the radiographic classification systems of Essex-Lopresti and Sanders are both useful and reliable prognostic tools to guide clinical decision in the treatment of these challenging fractures. Instead, there was no correlation of anatomical restoration of Böhler’s angle to better clinical results. For this reason, we believe it cannot have prognostic value as a measure to predict functional outcomes after surgery.

## Abbreviations

AOFAS: American Orthopaedic Foot and Ankle Society hindfoot scale
ASA: American Society of Anesthesiologists
BMI: Body mass index
CT: Computerized tomography
FFI: Foot Function Index
K-wire: Kirschner wire
LCD: Liquid crystal display
LET: Low-energy trauma
MCS: Mental component summary
MP: megapixel
MSF: Maryland Foot Score
OR: Odds ratio
ORIF: Open reduction and internal fixation
PCS: Physical component summary
PS: Percutaneous surgery
ROM: Range of motion
SF-36: Short form 36
US: United States
VAS: Visual analogue scale
